# The Experience of Women Affected by Polycystic Ovary Syndrome: A Qualitative Study From Iran

**DOI:** 10.5812/ijem.13612

**Published:** 2014-04-01

**Authors:** Fatemeh Nasiri Amiri, Fahimeh Ramezani Tehrani, Masoumeh Simbar, Ali Montazeri, Reza Ali Mohammadpour Thamtan

**Affiliations:** 1 Department of Midwifery and Reproductive Health, Shahid Beheshti University of Medical Sciences, Tehran, IR Iran; 2 Reproductive Endocrinology Center, Research Institute for Endocrine Sciences, Shahid Beheshti University of Medical Science, Tehran, IR Iran; 3 Mental Health Research Center, Health Metrics Research Centre, Iranian Institute for Health Sciences Research, Tehran, IR Iran; 4 Department of Biostatistics, Mazandaran University of Medical Sciences, Sari, IR Iran

**Keywords:** Polycystic Ovary Syndrome, Quality of Life, Qualitative Study, Experience

## Abstract

**Background:**

Polycystic ovary syndrome (PCOS) is the most common chronic endocrine disorder. It has significant and diverse clinical consequences including reproductive, metabolic, and psychological morbidities as well as predisposition to malignancies. It is unclear how women with PCOS experience symptoms of this syndrome.

**Objectives:**

The aim of this study was to clarify the dimensions and components of quality of life in iranian women with PCOS.

**Patients and Methods:**

This study was a qualitative study to explore and document perceptions of women with PCOS about their disorder and quality of life. Semi-structured interviews with open ended questions were conducted with 23 women with PCOS. The interviews were continued to reach data saturation. The study was conducted in the Reproductive Endocrinology Research Center of Shahid Beheshti University of Medical Sciences. All the interviews were recorded and transcribed. Constant comparative analysis of the data was conducted manually according to the Strauss and Corbin analysis method.

**Results:**

The study revealed that the most important factors affecting quality of life in women with PCOS were the role functioning items as well as physical, mental, emotional, cognitive, and social dimensions.

**Conclusions:**

Comprehensive cares concerning various mental, emotional, cognitive, and social dimensions of quality of life should be planned for women with PCOS.

## 1. Background

Polycystic ovary syndrome (PCOS) is one of the most common endocrine disorders in women during their reproductive years (1). The reported prevalence of PCOS among women of reproductive age varies according to its diagnostic criteria and is estimated to be 4% to 25% (2). Based on the Rotterdam criteria, its prevalence rate in Iran is 14.6% (3) and based on NIH criteria, the prevalence rate in Tehran is 8.5% (4). Although the exact etiology of PCOS is not well recognized, it is a hormonal disturbance accompanied by increased androgen and decreased function of reproductive system (5). In fact, PCOS is a multifactorial disorder caused by the interaction between genetic and environmental disturbances (6). PCOS can lead to various complications including obesity, metabolic disorders, cosmetic problems, infertility, uterine and cervical cancers, and psychological disorders (7-9). According to the previous researches, both physical health consequences and the emotional impact of PCOS have been ignored (10).

PCOS is a chronic disorder and due to its long-term complications, it can affect the quality of women’s life (11). PCOS and its influence on quality of life (QoL) is an issue that needs to be taken seriously as this syndrome affects many women across the world. Accurate measuring of the QoL is often hard. QoL was deﬁned by the World Health Organization (WHO) as an individuals’ perceptions of their position in life in the context of the culture and value systems in which they live and in relation to their goals, expectations, standards, and concerns (12). QoL in women with PCOS can be measured by general questionnaires; however, this type of questionnaires have little sensitivity to the unique aspects of PCOS on women’s health (13). Thus, it is argued that measuring QoL in patients with PCOS needs specific instruments. To our knowledge, there is only one specific questionnaire that measures QoL in this populations that has not been used in other populations (14). Furthermore, it has been criticized because of its low face and content validity (15). Therefore, there is a need for a comprehensive instrument to measure QoL in patients with PCOS. The physical aspects of PCOS have been explored in many studies (16-18); however, there are few studies concerning the experience of women affected by this disorder and their feelings were essentially neglected.

## 2. Objectives

The aim of this study was to explore the experiences of QoL in Iranian women with PCOS.

## 3. Patients and Methods

### 3.1. Participants and Data Collection

This was a qualitative study to explore and document perceptions of women with PCOS about their disorder and QoL. We conducted this study according to the conventional qualitative content analysis method. Qualitative content analysis is defined as a research method for the subjective interpretation of the content of text data through the systematic classification process of coding and identifying themes or patterns. The Process of content analysis includes the following:

- Preparation phase: becoming immersed in the data; in depth review of transcript for strives to make sense of the data and obtain a sense of whole.
- Organizing phase: open coding means that notes and headings are written in the text while reading it.
- Grouping phase: the lists of categories are grouped under higher order headings. The aim of grouping data is to reduce the number of categories by collapsing similar ones into broader higher order categories.
- Reporting phase: abstraction means formulating a general description of the research topic through generating categories (19, 20).

To collect data in-depth, interviews were conducted among a sample of patients with PCOS attending the Reproductive Endocrinology Research Center affiliated to Shahid Beheshti University of Medical Sciences between March and June 2011. After obtaining written informed consent, all the eligible women with PCOS who were referred to this center were interviewed at a privet room by using a semi-structured interview guide. The main researcher interviewed all the participants. All the interviews were audiotaped and were added to the material section. The women with PCOS who met our eligibility criteria and had willingness to participate in our study were recruited. They were included in the study if they had 18-40 years of age, met the Rotterdam criteria for diagnosis of PCOS (21), and concerned to participate and share their experiences. They were a heterogeneous group with various socioeconomic backgrounds and clinical phenotypes. The exclusion criteria included the coexisting illness, inability to read and speak Farsi, and having conditions similar to PCOS at presentation such as congenital adrenal hyperplasia. Interviews were conducted by the main researcher using a semi-structured guide questionnaire consisting of open-ended questions enabling respondents to fully explain their perceptions and experiences. All interviews were conducted in a private room and lasted 45-90 min (on average 75 min). To begin, each participant was asked to describe her individual perception about PCOS and her QoL. Each interview started with the same question: “We are trying to find out what it is been like for you living with PCOS. Therefore, what does having PCOS mean to you?” For each subsequent interview, further prompt questions were elicited from the themes identified from the transcripts. All interviews were taped and transcribed verbatim. During the interviews, notes were written regarding the nonverbal signals and were analyzed consecutively. Data saturation was achieved when no more codes were identified through the last three of interviews and when the emergent categories became coherent.

### 3.2. Data Analysis

Data collection and analysis were performed simultaneously according to the content analysis method. Meaning units, primary codes, sub categories, categories, and themes were applied to the data. During open coding, each transcript was reviewed by at least two authors and the data was reduced to codes. Differences in coding were resolved via discussions; codes that were found to be conceptually similar in nature or related in meaning were grouped into subcategories. In axial coding, the intent was to clarify how the emergent subcategories were related to preliminary categories. Analytical tools, including asking questions and making comparisons, were utilized to identify the properties of each concept. To determine the accuracy and integrity of data, acceptability, reliability, validity, and portability of data were evaluated using the Guba and Lincoln criteria (22). The accuracy of data were established by in-depth prolonged engagement with participants, selection of participants with various socioeconomic backgrounds and different clinical phenotypes. Results were also checked with some of the women with PCOS, who did not participate in the research and they confirmed the fitness of the results as well. In addition to the members of the research team, two experts in qualitative researches, who did not partici­pate in the research conducted a second review and con­firmed the fitness of the results as well. To increase data transferability, the researchers documented all of the steps followed in the research to facilitate repeating the study protocol in future researches. Results were also checked with a group of the women with PCOS who did not participate in the research to confirm the fitness of the results.

### 3.3. Ethics

The Ethics Committee of the Shahid Beheshti University of Medical Science approved the study protocol. Participants signed a written informed consent before the beginning of interviews.

## 4. Results

Overall, 23 in-depth interviews were conducted. Demographic characteristics of the study participants are presented in Table 1 and the major themes and sub themes of women’s QoL are shown in Table 2.

**Table 1.** Characteristics of the Study Participants ^a^

Characteristics	Values
**Age groups, y**	
18-24	8 (34.78)
25-34	13 (56.52)
35-40	2 (8.70)
**Body mass index, kg/m^2 ^**	
20-25	8 (34.78)
26-29	7 (30.44)
> 29	8 (34.78)
**Marital status**	
Married	15 (65.22)
Single	8 (34.78)
**Educational level**	
Primary	2 (8.70)
Secondary	15 (65.22)
Higher	6 (26.08)
**Occupational status**	
Housewives	10 (43.48)
Employed	9 (39.13)
Student	4 (17.39)
**Onset of PCOS**	
Onset at puberty	21 (91.30)
Post pubertal	2 (8.70)
**Main chief complain**	
Infertility	8 (34.78)
Hirsutism	3 (13.04)
Acne	1 (4.35)
Menstrual irregularities	4 (17.39)
Obesity	2 (8.70)
Recurrent abortion	3 (13.04)
Hair loss	2 (8.70)

^a^ Data are presented as No. (%)

**Table 2.** Major Themes and Subthemes of the Study

Themes
**Physical dimensions**
Obesity
Hirsutism
Hair loss
Acne
Menstrual disorder
Infertility
Ovaries full of cysts
Impaired general health
**Mental dimension**
Disappointment
Depression
Fear and anxiety
Introversion
Disgruntle
**Emotional dimension**
Low self-esteem
Feelings of shame and embarrassment
Poor sense of self and humiliation relative to peers
Being abnormal
Weakness
**Cognitive dimension**
Intellectual engagement
Poor concentration
Feeling inability in solving problems and planning
**Social dimension**
Lingering isolationism and withdrawal
Inability to express their problems
Blame by husband because of infertility
Limitations in participating in social gatherings
**Role functioning**
Gender roles
Loss of characteristics of femininity
Appearance of masculinity
Disorder of marital relationship

### 4.1. Physical Dimension

The experiences of the majority of participants showed feeling inconvenienced due to the effect of physical signs of this syndrome such as obesity, hirsutism, hair loss, acne, menstrual disorders, infertility, ovaries full of cysts, and impaired general health subjects. Therefore, most of them complained of infertility, obesity, and hirsutism. In this regard, a 28-year old employee stated, "Now I keep imaging I am pregnant, but this doesn’t happen, so I feel like i am incapable."

In addition, a 23-year-old housewife with a diploma, concerned about this syndrome, said, "The fertility problem is more important to me than the excess hair growth. I can solve the obesity problems, but not the excessive hair and fertility problem. I feel that if I can't bear a child, I will lose all sense of being a woman."

A 29-year old housewife said, "I’d like to have a child because my husband is very keen to have his son carry on his name."

The mean body mass index of the participants was 27.4 kg/m^2^. The majority of women with PCOS experienced weight-related problems including difficulty in losing weight, constantly fluctuating weight, and unexplained weight gain. Weight problems were described as having the most negative impact on QOL particularly on social and emotional well-being. In this regard, a graduate married 29-year-old woman said, ”Obesity makes lots of problems; I feel that many of my problems are rooted in obesity. I feel that my problems will be fade away if my obesity problems solve. Because I once had the experience of weight loss and my menstruation became regular and was at its exact time.”

In addition, a 24-year old housewife said with sorrow, “I see layers of fat protruding from each side of my body. Everyone who sees me says, ‘why you are so fat?’ “

Some women mentioned that their major problem was hirsutism. In this regard, a single 25-year old student said, "For me, primarily hirsutism is important because it affects people’s beauty."

In addition, a 27-year old single bachelor student employee said, “Hirsutism is so bad, because it is not like fuzz. I am waiting for my laser period to pass. During this period, I try not to look at this part of my face in the mirror.”

On the effects of this syndrome on self-perceived physical attractiveness, a 23-year-old single student said, "Beauty is very important for a woman. My hair loss is really bothering me; it is too severe. Excess body hair has also reduced my beauty. “

In addition, a 24-year-old housewife mentioned the impact of acne on herself, "Whenever I wear a dress with part of my body exposed, others look at my rashes; they zoom in on the rashes. Or when you go to a gathering, everyone looks at your face rash and asks, ‘Why do you have all those rashes on your face?’, and I don’t want to explain.” She then continued, "I could tolerate it if it was only the rash; my skin color darkens too. The rash scars remain on my face and make it full of spots. That’s why my hair is not important to me; the rashes do matter." 

Fewer women suffered from different physical symptoms and regarded the menstrual dysregulation more important than hirsutism. A 32-year old single graduate and unemployed woman said, “It is more important to me to treat my menstrual cycle because it is an inner factor. I can clean my unwanted hair.” she said about the reasons important of irregular menstruation that, “I always ask myself ‘why don’t I have regular menstrual cycle like others?’ ”

There were a small number of women with PCOS who felt unwell with ovaries full of cysts and felt pain and discomfort especially after hard physical exercise or a fast walking. Some of these women recognized the feeling of pain and discomfort under their belly as the reasons of lack of exercise. In this case, a 39-year-old married woman said, “I went to gym but I felt so bad so that I had to call my friend to come. She took my arm and took me home. Therefore, I was ashamed because I bothered others. Finally, I gave up exercising and never went back to gym; I feel the same as I walk quickly.”

The majority of women with PCOS had good general health, but some women had sleep disorders, fatigue, low energy, and feeling unwell. In this case, a 26-year-old single woman said, “I sleep badly; I occasionally wake up during my sleep time. Most of the nights, I just can sleep about 3-4 h; my best time record is 3-4 h, and after waking up in the morning, I don’t feel that I have slept last night because I feel so sleepy again. If I could, I would sleep all day long!”

Some of these women view themselves different from their peers in terms of energy and freshness. Hereon, a 26-year-old single woman said, “I think that I am very different from others. My peers are very energetic and stirring, but I get tired very early.”

### 4.2. Mental Dimension

In mental dimension, experience of the most of the participants included depression, disappointment, fear and anxiety, introverted, and disgruntled. The majority of women suffered from PCOS were satisfied with their physical health but they felt that they had different mental health from others. In this regard, a 28-year-old married and employed patient said, “Health is not just the absence of illness or pain. In my opinion, the psychological aspect of this syndrome is more important than the pain.” 

In addition, a 25-year-old female student stated, “At first, it was just hirsutism and I still had not visited the doctor; but then I saw my hair and it made me nervous. I wondered why there is so much hair on my face and this made me nervous. I was in my room all the time, I felt depressed and disappointed.”

With regard to the introverted manner, a 23-year-old married woman said, “I just try not to talk too much with others because I think after talking about this issue, it gets more complicated. However, I can prevent this trauma if I do not talk much and try to stand and wait more. I try to limit my relationships and I don’t communicate openly.”

### 4.3. Emotional Dimension

In emotional dimension, the experience of participants included low self-confidence, feeling the shame and embarrassment, feeling humiliation and inferiority towards peers, and feelings of weakness and abnormality. 

In this regard, a 25-year-old single woman said, “This hirsutism is the reason of my low self-confidence. Every time I remove these hairs, they grow again quickly. I must always select my cloth in a way to cover my unwanted hair, because having much amount of hair makes me shameful. When I see another woman without hair, I just compare myself with her. I think how fortunate she is without hair and how different I am from her. I am so unusual because I don’t have soft and tender hair like others.”

In this case, a 28-year-old employed and bachelor graduated woman stated, “I feel that I am so weak because of this syndrome. I find it painful. People do not have any pain with this syndrome, but there are many emotional and mental problems. Sometimes, I sit down, stare, and think about this matter for an hour; ‘what will happen then?’ Is there any treatment or it is limited like many other things?”

### 4.4. Cognitive Dimension

With respect to the cognitive dimension, the experiences of participants included intellectual engagement, poor concentration, and feeling inability in solving the problems and planning. In this case, a 27-year-old married and unemployed woman said, “My hirsutism always comes into my mind; I think that I must remove my hair every other day. The hair under my chin usually grows too fast, it bothers me, and it will appear again after two days. Black spots appear below them.”

In addition, a 33-year-old employed woman complained of her inability to solve her problems and said, “This syndrome causes problems that make me to seek refuge in restaurants and fast foods. My mood after the loss of a pregnancy is very weak and my strength has declined."

In case of problem in planning, a 26-year-old married graduate woman said, “Because of irregular menstrual periods, this disorder impairs body systems. It mixes up all the plans; for example, we would go to travel since I know I’m not in my menstrual period during this time, but I would be, without planning.”

### 4.5. Social Dimension

Another experience of women with PCOS, which was highly repeated, was the reduction of interpersonal and social interaction and inappropriate reaction of society towards the side effects of this syndrome. In this regard, a 23-year-old married woman stated, “From physical aspect, I think I am much healthier; but I feel I am not social at all. I have lack of interaction and communication with other people; for example, I am not smart in communication.”

Moreover, a 25-year-old single participant said with tension and resentment of other people’s reaction in society, “I gain weight since I am suffering from this disorder. Last time I visited my doctor, a woman asked me, ‘are you single?’ After I answered, ‘yes’ she said, ‘a single girl shouldn’t have such a big belly’, and I got so embarrassed. Although I put on the veil, she could see my big belly.”

In addition, a 23-year-old married woman said with sadness, “Sometimes I hear something about hirsutism or infertility and I think if other people understand you and ignore your problem, it would be easier to cope with this problem; otherwise it would be so hard.”

A 26-year-old single woman said, “Recently, I want to be alone. Due to my severe hair loss, I prefer to be alone. I got a little depressed because I see my peers with bushy hair that I do not have. I got depressed when I compare myself with them; then, I prefer to stay at home.”

### 4.6. Role Functioning

Based on the data analysis, role functioning of the QoL in women with PCOS included gender-related role, loss of feminine characteristics and incidence of masculine characteristics, and disruption in marital relationship. The extracted theme, main categories, and sub-categories are shown in Table 2.

Findings of this research show that the majority of women with PCOS have considered themselves unattractive and had a negative self image because of the side effects of this syndrome; this is why they felt ashamed and embarrassed. In this regard, a 29-year-old graduate married woman said with sadness, “I am not satisfied with my appearance because obesity doesn’t let me wear the cloth I like. I am embarrassed in parties and wedding celebrations.”

Some women with PCOS were unsatisfied with their role of being a woman and feel displeasure. In this regard, a 40-year-old housewife said, “Men are more comfortable, I was more comfortable if I wasn’t a woman. My husband argues with me so much. He said, ‘you are infertile’, and therefore, he wants separation. ''My husband has constantly called me” infertile". It is now 10 years that he doesn’t like to come home' 

The other experience of these women was the effect of this disorder on femininity feeling and incidence of masculine characteristics. A 23-year-old married housewife said, “I feel more masculine, but my husband really affected me after marriage. For example, he always told me that ‘you mainly behave manly, it seems like you are not a woman and your masculine tempers are more.’ My husband and I had different problems because he always said I have manly features.” She continued, “I feel it affects the way I dressed, because I preferred to wear shirts and pants more.”

Some women were satisfied with their marital relationship, but others were embarrassed because of sexual dysfunction and cold tempered. In this regard, a participant stated, “I feel I don’t like my husband’s smell like pregnant woman. In addition, I feel it is really a farce. I do not have any desire to do that; nothing is irritating for me. I just want this relationship to end and finish my pain soon. I even went to doctor but I didn’t have any infection.”

In addition, a 23-year-old graduate housewife said, “My hair was bristle and my husband mentioned it at first; but after a while, he didn’t mention it any more. It was important to me to be comfortable in front of my husband. It is really hard, because after marriage man expect different things; perhaps the reason of his reluctance [to intercourse] was my hirsutism, because I had too much hair. Whenever I took a shower, I had to spend about two hours to remove my hair. I was tired since it took so much time. After three days, they grew again as if I had done nothing. I didn’t pay more attention because it was hard and time consuming, but when I saw the difference in my relationship with my husband and it got better, I cared more and more.”

Most of the women with PCOS had this feeling but some of them had different feeling. A few of them were satisfied with their sexual performance and believed that this syndrome has not had any impact on their sexual attractiveness. For instance, a 28-year-old married and employee female said, “I feel that this syndrome has not affected my sexual function. My libido is better; so is my sexual desire. I am not a cold woman.”

## 5. Discussion

This qualitative study explored the experiences of Iranian women with PCOS. The novel findings from this study related to the fact that we found a new dimension as role functioning in women’s life that was not identified in previous research on the topic; this syndrome is highly destructive to the ‘feminine’ norms.

Considering the life experiences of women with PCOS, many factors can affect the quality of their life with physical, psychological, emotional, social, and cognitive influences. Due to development of this syndrome, their beauty was impaired by obesity, hirsutism, hair loss, and facial acne. In addition, resulted menstrual disorders and infertility had impaired their femininity feeling, had made them ashamed, and had decreased their self-confidence as they considered themselves inferior to other women.

The assessment of QoL in women with PCOS helps in understanding the experiences of women and deeper understanding of their illness. In addition, it would help health care providers in providing health services and educating these women to cope with their disorder and enhance their self-confidence.

In study of Weiss et al., the physical side effects that could influence the QoL in women with PCOS including hirsutism, android alopecia (male pattern baldness), and weight gain were assessed. This study shows that symptoms of PCOS decreased the QoL in women and due to changes caused by this syndrome, they experienced depression, disappointment, and shame as well as considering themselves as imperfect women (23). 

Other factors affecting the QoL in these women are psychological, emotional, and cognitive problems. Teede et al. in a review study indicated that PCOS was a frustrating experience for women with negative effects on their psychological and mental health and greatly reduced their QoL (24). Himelein and Thatcher in their study stated that women with PCOS often experienced the feeling of depression, isolation, anxiety, and frustration (25). The psychological distress and decrease in QoL might be seen even in women who have received medical care (26).

Based on experiences of participants, other factors that affected their QoL were reduction of interpersonal and social relationships and people’s inappropriate reaction towards the side effects of this syndrome. The majority of the women participated in this study stated that the symptoms of this syndrome had a negative impact on their body image and it made them feel ashamed and low self-confidence. This finding is consistent with other studies (27, 28). In this regard, Trent et al. found that most common symptoms of PCOS such as menstrual irregularities, obesity, male pattern of body hair growth, acne, and other skin problems played a major role in impaired body image, self-confidence, and QoL (29). Hopwood et al. explained for the first time that body image was an important goal in evaluating the QoL in the treatment of cancers that cause major changes in patient’s appearances due to surgery, delayed effects of radiotherapy, or side effects of systemic medications (30). The loss of feminine characteristics and the appearance of masculine features were the effects of PCOS that repeatedly mentioned by the study participants. Likewise, in a study by Weiss et al., one of the major concerns of women with PCOS was an assault on their feminine characteristics due to hirsutism, alopecia (androgenetic baldness), and weight gain (23). In this study, women considered themselves physically less than other women and felt disappointed, angry, and embarrassed because of the syndrome-induced changes. In a qualitative study by Synder B, women with PCOS described the feelings of being different from others and hirsutism had negatively affected their sense of femininity (31). Boomsma et al. had reported that the syndrome led to the deepening of patients' voice due to increased androgens, as well as to breasts shrinkage and increased muscle mass in the body; the findings that are in agreement with experiences of Iranian women (32).

Findings of this study showed that the majority of women with PCOS had experienced problems in their sexual performance because of the effects of the symptoms on their physical appearance. Elsenbruch et al. indicated that there were different factors that could affect the quality of sexual performance in women with PCOS. Changes in body dimensions and physical beauty (because of hirsutism, obesity, acne, and alopecia) as well as imbalance of sexual hormones could lead to reduction in QoL and sexual performance (26).

in addition, Hahn et al. reported that women with PCOS believed that their sexual partners were less satisfied with their sexual lives and considered themselves unattractive (33). Coffey et al. concluded that the sexual performance of women with PCOS might be affected by physical factors caused by this syndrome (34). Women with PCOS also thought that their partners found them less sexually attractive (35). Sexual self-worth seems to be lower in women with PCOS (36, 37). Higher body image disturbance is associated with sexual avoidance (38).

In our study, the majority of women reported fear of infertility at present and in the future. In agreement with our study, in a cross-sectional study Trent et al. reported that girls with PCOS were 3.4 times more likely to be “worried about their ability to become pregnant in the future” in comparison to the healthy adolescents (68% vs. 41%) with controlling for age (38). Infertility was found to have negative effects on marital relations as one of the spouses requested divorce or separation (39). Our data showed that infertility affected the relationships of the couples because the infertile spouse felt guilty and considered separating or getting divorced for not being able to give a child to his partner. 

One limitation to this study was the nature of the sample, which consisted of volunteers recruited from Reproductive Endocrinology Research Center. It is also possible that the experiences reported by volunteer participants might have been more positive than those who chose not to be interviewed. Limitations of this study were overcome by the use of purposive sampling as the sample was more heterogeneous demographically, especially in educational attainment and PCOS manifestations. The small sample is not necessarily representative of all Iranian women with PCOS. To explore how different cultural contexts shape experiences of woman with PCOS to view and percept their symptoms, further researches with a more diverse sample using the findings of cultural studies of QoL are recommended.

The final conclusion is that PCOS is a heterogeneous disorder and its main symptoms not only affect women’s beauty and undermined their QoL, but also decrease their femininity feeling and make them believe they are “inferior” in comparison with other women. This is why psychological, emotional, and cognitive dimensions in women with PCOS were disturbed and the feeling of humiliation and shame had been prevailed in their interpersonal and social relationships. As a result, a multidimensional and dramatic effect of this disorder cultural-based approach through health education and counseling is recommended. In this way, beside treatment of symptoms and extended effects of this disorder, we can improve the QoL of these women too.
